# Role of Alveolar Macrophages in Chronic Obstructive Pulmonary Disease

**DOI:** 10.3389/fimmu.2014.00435

**Published:** 2014-09-10

**Authors:** Ross Vlahos, Steven Bozinovski

**Affiliations:** ^1^Department of Pharmacology and Therapeutics, Lung Health Research Centre, The University of Melbourne, Parkville, VIC, Australia

**Keywords:** alveolar macrophage, chronic obstructive pulmonary disease, efferocytosis, lung inflammation, oxidative stress, resolution

## Abstract

Alveolar macrophages (AMs) represent a unique leukocyte population that responds to airborne irritants and microbes. This distinct microenvironment coordinates the maturation of long-lived AMs, which originate from fetal blood monocytes and self-renew through mechanisms dependent on GM-CSF and CSF-1 signaling. Peripheral blood monocytes can also replenish lung macrophages; however, this appears to occur in a stimuli specific manner. In addition to mounting an appropriate immune response during infection and injury, AMs actively coordinate the resolution of inflammation through efferocytosis of apoptotic cells. Any perturbation of this process can lead to deleterious responses. In chronic obstructive pulmonary disease (COPD), there is an accumulation of airway macrophages that do not conform to the classic M1/M2 dichotomy. There is also a skewed transcriptome profile that favors expression of wound-healing M2 markers, which is reflective of a deficiency to resolve inflammation. Endogenous mediators that can promote an imbalance in inhibitory M1 vs. healing M2 macrophages are discussed, as they are the plausible mechanisms underlying why AMs fail to effectively resolve inflammation and restore normal lung homeostasis in COPD.

## Introduction

Macrophages are essential for pulmonary host defense through their capacity to survey the exposed airways and regulate innate and adaptive immunity. The pulmonary macrophage system consists of several different populations that are found in anatomically distinct compartments, including the airways, alveolar spaces [alveolar macrophages (AMs)], and resident lung tissue. AMs constitute over 90% of the pulmonary macrophage population (1) and have traditionally thought to originate from the bone marrow (2). More recently, it has been shown that although AMs originate from fetal blood monocytes within the first week of life via GM-CSF dependent mechanisms, maintenance of this population during homeostasis is dependent on their self-renewing capacity (3). Once, developed, this lung macrophage pool is long-lived in humans (2) in the absence of an inflammatory insult. This finding has been replicated in mice, where there was approximately 40% turnover of AMs over 1 year in the absence of an insult (4). Hence, the macrophage lung population, particularly during the steady state, is primarily sustained by the self-renewal of pulmonary AMs through local proliferation (5). The local proliferation of AMs during homeostatic repletion is dependent on both GM-CSF and CSF-1, with no dependence on IL-4 signaling (6). Both GM-CSF and M-CSF also control the proliferation and survival of AMs (7). The differentiation of AMs is particularly dependent on GM-CSF, where the leukocyte growth factor regulates essential functions including phagocytosis and surfactant catabolism through the PU.1 transcription factor (8). This is consistent with a unique airway environment that is associated with high oxygen tension and high levels of GM-CSF.

Resident AMs are constantly encountering inhaled substances due to their exposed position in the alveolar lumen. AMs are considered to be major effector cells in innate host defense against inhaled irritants by virtue of their phagocytic ability (9). Therefore, it is vital that resident AMs are kept in a relatively quiescent state with active suppression of inflammation in response to harmless antigens to prevent collateral damage to lung tissue (10). Although AM exhibit microbicidal, tumoricidal, and parasiticidal activities (9, 11), they are functionally less responsive than tissue-resident macrophages. Relative to tissue macrophages, they display reduced phagocytic capacity, reduced respiratory burst, and a diminished capacity to present antigen to T cells (12–14). Under homeostatic conditions, AMs are closely associated with alveolar epithelial cells (AECs), and this in turn induces the expression of epithelial restricted αvβ6 integrin that binds and activates latent TGFβ (15). TGFβ can inhibit macrophage activation that is implicated in alveolar wall destruction (15). Upon recognition of antigens by TLRs, the rapid induction of actin polymerization promotes AMs detachment from AECs (16). The subsequent production of proteases by activated AMs then activates latent TGFβ, thereby reinstating AMs to their resting state (16). This illustrates an intricate mechanism of microenvironmental macrophage specialization to keep the macrophage response in check. AMs can also produce TGFβ, which suppresses T cell activation and has been shown to promote the emergence of T regulatory cells (17).

## Role of AMs in Innate Host Defense

In contrast to the self-renewing capacity of AMs during the steady state, inflammatory and infective insults dramatically change the dynamics of local lung macrophages. Using chimeric mice, parabiosis, and adoptive cellular transfer models, it has been established that bone marrow-derived blood monocytes replenish the AM pool during lethal irradiation (3). This is context specific, as inoculation with influenza in mice, which dramatically depletes resident AMs in a strain specific manner, led to restoration of this population through self-renewal proliferative mechanisms (6). This infectious model also promotes the substantial recruitment of blood monocytes; however, the fate of monocyte-derived macrophages has yet to be established in this setting. Using a similar approach, LPS has been shown to restore lung macrophage numbers through both local proliferation of resident macrophages and maturation of recruited blood monocytes (18). Mouse blood monocytes can be subdivided according to differential expression of chemokine receptors and adhesion molecules that are involved in cell recruitment. CCR^2+^Gr1^hi^CX_3_CR1^lo^ is actively recruited to inflamed tissues by virtue of their recognition of CCL2 (also known as MCP-1) (19). Mouse monocytes that are CCR^2+^Gr1^hi^CX_3_CR1^lo^ are classified as pro-inflammatory but share morphological characteristic and chemokine receptor patterns with the classical human monocytes (CD14^hi^CD16^−^CX_3_CR1^lo^) (19). Monocyte-derived macrophages can acquire distinct morphological and functional properties as directed by the immunological microenvironment.

Alveolar macrophages coordinate antimicrobial defenses through expression of receptors for immunoglobulin (F_c_R), complement, β-glucan, mannose, and several types of scavenger receptors that together facilitates phagocytosis (20). AMs generate reactive nitrogen and oxygen intermediates involved in macrophage-mediated defense against microbial infection (21, 22). Surfactant protein A augments pathogen killing by AMs by stimulating phagocytosis and production of reactive oxygen–nitrogen intermediates (23). In addition, AMs can initiate recruitment of inflammatory cells from pulmonary vasculature into the alveolar space. There are a number of studies that implicate AMs as central effector cells in the production of pro-inflammatory cytokines, which initiate the early phase of neutrophil influx in response to acute lung injury caused by bacterial products (24, 25). More recently, selective targeting strategies that ablate different monocyte/macrophage populations have identified an important role for peripheral blood monocytes. Ablation of CCR2^hi^ monocytes significantly reduced indices of acute lung injury (26). It is plausible that AMs may actually play a role in limiting neutrophil influx by controlling MCP-1 production through AECs (27). Furthermore, in a murine model of pneumococcal pneumonia, AMs depletion resulted in a failure to modulate the inflammatory response with increased levels of pro-inflammatory cytokines (28). AMs are also central regulators of the resolution of inflammation through their ability to engulf apoptotic neutrophils during the resolution phase (28, 29). The active phagocytosis of dying cells by macrophages may also lead to the induction of anti-inflammatory or suppressive properties in macrophages as shown by the inhibition of IL-1β, IL-8, TNFα, and GM-CSF production (30).

Alveolar macrophages are also indispensable for the clearance of influenza infection in a viral strain-dependent manner in mice. Here, it was established that the depletion of airway macrophages was associated with more severe lung injury following inoculation with BJx109, which is a viral strain that infects macrophages with high efficiency (31). This is in contrast to the highly virulent PR8 strain that poorly replicated in airway macrophages, suggesting that avoidance of AM engagement may contribute to the virulence of influenza strains (31). There is also the important clinical complication of secondary bacterial infections following a significant viral event, where AM function is likely to be compromised. In murine models of secondary bacterial infection, the initial depletion of AMs as a consequence of influenza infection rendered the host susceptible to *Streptococcus pneumonia* (Spn) colonization and systemic invasion (32). The repletion of resident AMs occurred 2 weeks after influenza, which resulted in the re-establishment of early innate host protection to Spn (32). This AM replenishment phase may represent a window of opportunity for opportunistic respiratory pathogens such as Spn that take advantage of this immunocompromised state. Interferon-γ production during the recovery phase of a viral infection can also inhibit lung anti-bacterial defenses. Mechanistically, it was shown that viral-induced production of inteferon-γ caused downregulation of the scavenger receptor MARCO, and neutralization of interferon-γ prevented secondary pneumococcal infection (33). Using MARCO-deficient mice, it has been established that expression of this scavenger receptor on AMs is critical for efficient clearance of Spn from the lungs (34).

## Role of AMs in Chronic Obstructive Pulmonary Disease

There is a large body of evidence implicating AMs in the pathogenesis of Chronic Obstructive Pulmonary Disease (COPD). COPD is a major global health problem and has been predicted to become the third largest cause of death in the world by 2020 (35). Cigarette smoking is the major cause of COPD and accounts for more than 95% of cases in industrialized countries (36), but other environmental pollutants are important causes in developing countries (37). COPD is “a disease state characterized by airflow limitation that is not fully reversible.” The airflow limitation is usually progressive and associated with an abnormal inflammatory response of lungs to noxious particles and gases (38). COPD encompasses chronic obstructive bronchiolitis with fibrosis and obstruction of small airways and emphysema with enlargement of airspaces and destruction of lung parenchyma, loss of lung elasticity, and closure of small airways. Most patients with COPD have all three pathologic conditions (chronic obstructive bronchiolitis, emphysema, and mucus plugging), but the relative extent of emphysema and obstructive bronchiolitis within individual patients can vary.

Studies have highlighted that macrophages play a pivotal role in the pathophysiology of COPD (39). There is a marked increase (5- to 10-fold) in the numbers of macrophages in airways, lung parenchyma, bronchoalveolar lavage fluid (BALF), and sputum in patients with COPD (40, 41). A morphometric analysis of macrophage numbers in the parenchyma of patients with emphysema showed a 25-fold increase in the numbers of macrophages in the tissue and alveolar space compared to smokers with normal lung function (42). There is a positive correlation between macrophage numbers in the airways and the severity of COPD (43). In addition, a pathological role for macrophages has been demonstrated, as the depletion of lung macrophages conferred protection against the development of emphysema in an experimental model of COPD (44). Macrophages are activated by cigarette smoke and other irritants to release inflammatory mediators. AMs also secrete elastolytic enzymes (proteases), including matrix metalloprotease (MMP)-2, MMP-9, MMP-12, cathepsin K, L, and S in response to irritants and infection, which together are responsible for destruction of lung parenchyma (36). In patients with emphysema, there is an increase in BALF concentrations and macrophage expression of MMP-1 and MMP-9 (45). There is an increase in activity of MMP-9 in the lung parenchyma of patients with emphysema (46). AMs from smokers with normal lung function express more MMP-9 than those from non-smoking healthy subjects (47), and there is an even greater increase in cells from patients with COPD, which have enhanced elastolytic activity (48).

Chronic obstructive pulmonary disease subjects can also be very susceptible to bacterial colonization (49, 50) and exacerbations that are commonly caused by respiratory infections of viral and/or bacterial etiology (51). The frequent exacerbator phenotype has been reported, which is associated with a poorer quality of life and increased systemic inflammation (52). Impaired AM function is central to high colonization rates and increased susceptibility to exacerbations observed in COPD. Chronic cigarette smoke exposure is a major cause of COPD, which markedly depletes intracellular GSH stores (53, 54). Oxidative stress leads to disruption of GSH metabolism, which is considered as a key susceptibility feature of lung diseases (55). Excessive oxidative stress is particularly deleterious to AM function, leading to a deficiency in phagocytosis of bacteria (56) and efferocytosis of apoptotic cells (57). Treatment with anti-oxidants such as procysteine can significantly improve efferocytic function of AMs isolated from experimental models of COPD (58). Impaired AM-mediated efferocytosis in COPD can be particularly damaging in COPD as neutrophils are persistently recruited into the airways. Cigarette smoke impairs clearance of apoptotic cells through oxidant-dependent activation of RhoA (59) and inhibition of Rac1 (60), leading to defective actin polymerization normally required for efficient efferocytosis. The inability to efficiently remove exhausted neutrophils has damaging implications in COPD as accumulation of necrotic neutrophils can lead to the indiscriminate release of granule protease pools including neutrophil elastase. Neutrophil elastase localizes to lung elastic fibers in emphysematic patients and degrades extracellular matrix components (61) and can promote the release of mucins through epidermal growth factor receptor (EGFR)-dependent mechanisms (62). EGFR transactivation can also augment inflammatory responses initiated by rhinovirus infection (63). Reactive free radicals also impair clearance mechanisms by directly causing cytoskeletal instability and carbonyl modification of pseudopodia (64–66). Macrophages also interact with carbonyl-adduct modified extracellular matrix proteins, which impair their ability to clear apoptotic neutrophils (67).

In addition to oxidative post translational modification of the host phagocytic machinery, the complex milieu within COPD airways can alter the phenotype of highly plastic airway macrophages. It is becoming increasing clear that different macrophage subpopulations exist in the inflamed lung. Although the existence of such populations is implicated in COPD, the importance of these subpopulations is unknown (68). The ongoing characterization of disease-associated macrophages clearly demonstrates that they do not conform into the classic M1/M2 dichotomy and it is likely that the inflammatory environment of the COPD airways drives development of both M1 and M2 macrophages (58, 69). Indeed, it has been shown that iNOS and arginase activity are concurrently elevated in COPD airways. Specifically, elevated iNOS expression has been observed in AMs of COPD patients, which increased with severity of disease and during exacerbations (70–72). Of interest, levels of exhaled nitric oxide (NO) are not elevated in stable COPD, which may be consequential to increased production of superoxide that reacts with NO to generate the highly reactive nitrogen species, peroxynitrite (73). In addition, arginase-1 is increased in cigarette smoke exposure models (74), which will reduce l-arginine availability to iNOS. Reduced l-arginine bioavailability also stimulates iNOS to simultaneously produce NO and superoxide, which facilitates rapid formation of peroxynitrite (73). Consistent with the concurrent existence of iNOS and arginase expressing macrophages in COPD, nitrotyrosine (product of peroxynitrite) levels have been shown to be increased in sputum macrophages of COPD patients, which are negatively correlated with their lung function (75). Hence, the relative ratio of iNOS expressing M1 macrophages and arginase expressing M2 macrophages will be particularly important to the oxidative/nitrosative state of the lung.

The relative balance between these polarization states can in turn, have a profound impact on disease progression [reviewed in Ref. (76)]. There is evidence for the reprograming of AMs as a consequence of chronic smoke exposure that is associated with the induction of a unique set of genes including MMP12 (77). There is also evidence for the transcriptional skewing of AMs toward an M2 gene profile in smokers with normal lung function that was more evident in smokers who had progressed to develop COPD (78). In this study, they also demonstrate the progressive downregulation of M1 genes (78), which would appear paradoxical to the observation of increased expression of pro-inflammatory mediators in COPD. Hence, there is a need to better define the relative contribution of M1 vs. M2 macrophages as there is emerging evidence that both populations do concurrently exist in COPD airways. Another important consideration in COPD is the interaction between macrophages and T cell subsets. Although it has been broadly stated that T cells control the polarization state of macrophages, macrophages can also potently regulate T cell biology (79, 80). Since T cell subsets including CD8^+^ T cells, T_H_17, and iBALT formation are implicated in COPD pathology, the role of macrophages in regulating T cell biology remains to be elucidated.

The accumulation of M2 skewed airway macrophages may be reflective of deficient resolution processes that normally switch off inflammation and restore lung homeostasis. Since the stimulation of non-phlogistic phagocytosis is essential to resolution of inflammation, the oxidant-dependent impairment of efferocytic clearance of damaged tissue may maintain M2 macrophages in COPD. The induction of CD163 is commonly recognized as a marker for M2-alternatively activated macrophages involved in wound-healing (81, 82) and CD163 positive macrophages are highly prominent in the BAL compartment of current and ex-smokers with COPD (83). CD163 may constitute a major defense mechanism to protect the lung as it functions as a scavenger receptor, which promotes degradation of HbHp complexes and signaling that induces expression of heme-oxygenase-1 (HO-1) (82). The persistence of HO-1 in COPD airways (84) is consistent with an environment where there is excessive oxidative stress and a deficiency in the resolution of inflammation. The mediators that induce expression of M2 markers in COPD have not been comprehensively characterized. Interleukin-10 (IL-10) is a potent inducer of CD163 expression in human monocytes (85); however, there is some data to suggest that the level of IL-10 positive macrophages is reduced in COPD (86). An alternate mediator that has been shown to potently induce expression of CD163 in human monocyte-derived macrophages is serum amyloid A (SAA) (87). SAA is a major acute phase reactant that has now been shown to be expressed in COPD lungs, where its levels correlated with neutrophilic inflammation (88). SAA is known to target the ALX/FPR2 receptor and oppose the actions of pro-resolving ligands such as LipoxinA_4_ (88–90), which normally stimulate non-phlogistic clearance pathways. In addition to CD163, SAA can also stimulate the expression of the T_H_17 polarizing cytokines, IL-6, and IL-1β in monocyte-derived macrophages, and neutralization of IL-17A expression suppressed neutrophil airway inflammation stimulated by SAA (91). Hence, the persistence of host defense mediators such as SAA may maintain alternative macrophage populations in COPD airways that not only express M2 markers of wound repair, but also markers of acute inflammation.

## Conclusion

In this review, the origin and maintenance of AMs and their essential role in innate immunity to respiratory pathogens are discussed. There is emerging evidence for the self-renewal of AMs and lung tissue macrophages through mechanisms dependent on GM-CSF and CSF-1 signaling. The role of peripheral monocytes in replenishing lung macrophages appear to be context specific, as radiation-induced lung injury stimulates monocytic replenishment, whereas influenza infection replenishes AMs through local proliferation of resident macrophages. In diseases such as COPD where there is an accumulation of airway macrophages, the relative contribution of monocyte versus local proliferation of mature AM populations remains to be determined. Lineage tracing of monocytes/macrophages in experimental models of COPD will inform on the origin of macrophages associated with disease pathology.

**Figure 1:**
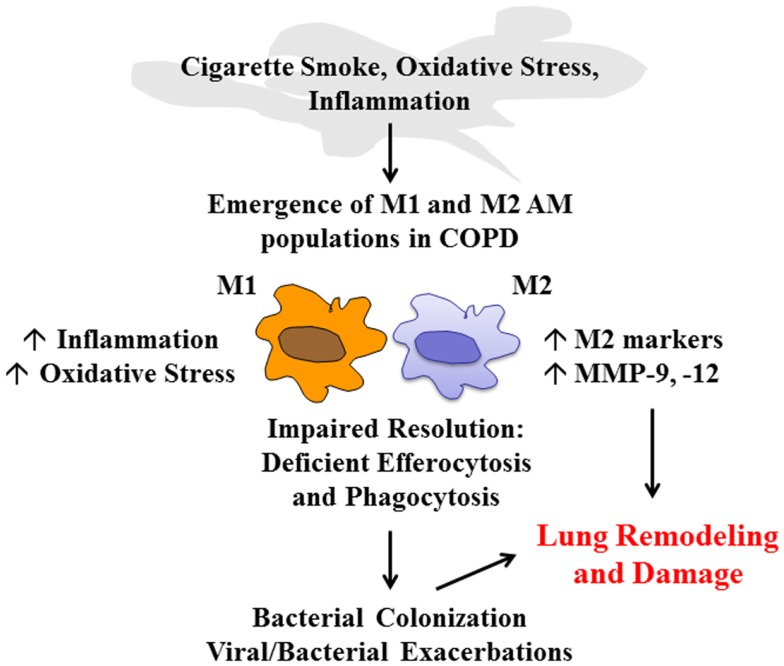
**COPD alveolar macrophage**. Cigarette smoke, oxidative stress, and the airway inflammatory microenvironment have a direct effect on alveolar macrophage (AM) phenotype in COPD that leads to the emergence of M1 and M2 populations. The ratio of these macrophages will govern the pathological processes in COPD. M1 macrophages will further drive inflammation and oxidative stress. Excessive oxidative stress impairs resolution mechanisms including macrophage-mediated phagocytosis and efferocytosis, which leads to colonization and exacerbations in COPD. In addition, the emergence of M2 macrophages can contribute to deleterious lung remodeling/damage through increased expression of M2-related genes and excessive protease (MMP-9, -12) production.

Alveolar macrophages coordinate the efficient clearance of inhaled irritants and microbes to resolve inflammation. In addition, AMs display efferocytic activity to clear damaged tissue and cells following injury and infection. In COPD, the persistence of inflammation and the inability to efficiently clear damaged tissue and exhausted immune cells such as neutrophils may be due to excessive oxidative stress that impairs the phagocytic capacity of AMs. Airway macrophages in COPD also display a unique phenotype that is associated with the induction of M2-related genes, which are likely to be upregulated in response to local tissue damage (Figure 1). The maintenance of this subpopulation may also contribute to deleterious remodeling in COPD. Thus, there is a need to better characterize distinct AM populations present in COPD and their relative contribution to disease pathology, as their highly plastic nature offer a therapeutic opportunity to reprogram macrophages to facilitate restoration of lung homeostasis.

## Conflict of Interest Statement

The authors declare that the research was conducted in the absence of any commercial or financial relationships that could be construed as a potential conflict of interest.
